# Investigation on returning to work in liver cancer survivors in Taiwan: a 5-year follow-up study

**DOI:** 10.1186/s12889-021-11872-9

**Published:** 2021-10-12

**Authors:** Shih-Wei Yang, Wei-Liang Chen, Wei-Te Wu, Chung-Ching Wang

**Affiliations:** 1 Department of Orthopedic, Tri-Service General Hospital; and School of Medicine, National Defense Medical Center, Taipei, Taiwan, Republic of China; 2 Division of Family Medicine, Department of Family and Community Medicine, Tri-Service General Hospital; and School of Medicine, National Defense Medical Center, Taipei, Taiwan, Republic of China; 3 Department of General Medicine, Tri-Service General Hospital; and School of Medicine, National Defense Medical Center, Taipei, Taiwan, Republic of China; 4 Division of Geriatric Medicine, Department of Family and Community Medicine, Tri-Service General Hospital; and School of Medicine, National Defense Medical Center, Taipei, Taiwan, Republic of China; 5 National Institute of Environmental Health Science, National Health Research Institutes, Miaoli, Taiwan, Republic of China; 6 Division of Environmental Health & Occupational Medicine, Department of Family & Community Medicine, Tri-Service General Hospital, National Defense Medical Center, Taipei, Taiwan, Republic of China

**Keywords:** Liver neoplasms, Cancer survivors, Return-to-work, Workers

## Abstract

**Background:**

Primary liver cancer is the fifth most common malignancy and limits patients’ quality of life and working ability. Return to work after cancer treatment is an important step in social recovery. In addition, return to work represents the recovery of financial ability and improvements in self-confidence. The purpose of this article is to discuss the relationship between return to work and various covariables in workers with liver cancer.

**Methods:**

The national registry cohort study collected adult workers newly diagnosed with liver cancer from 2004 to 2010 in Taiwan. There were 2451 workers included in our study. Primary liver cancer was diagnosed by using the International Classification of Diseases for Oncology code. Return to work after liver cancer survival was determined as returning to the same work or reemployment within five years after cancer diagnosis. The associations between independent variables and return to work were analyzed by Cox proportional hazard models.

**Results:**

Workers who underwent surgery were more likely to return to work not only in the 2nd year but also in the 5th year. A lower survival rate was noted in the non-return-to-work group (*p* < 0.001) among all patients with liver cancer. The completely adjusted model identified that the rate of return to work was related to all-cause mortality with a hazard ratio of 0.244 (95% Confidence Intervals: 0.235–0.253).

**Conclusions:**

Our study indicated the impacts of treatment on the return to work of liver cancer survivors. In addition, in patient with liver cancer, return to work had positive effect on the survival rate.

**Supplementary Information:**

The online version contains supplementary material available at 10.1186/s12889-021-11872-9.

## Background

Cancer is a major cause of morbidity and mortality in twenty-first century. Cancer impacts not only physical capacity but also mental health of patients. Cancer decreases not only working capacity but also quality of life. Due to early detection and the development of treatment options, the prognosis and outcomes of cancer patients have improved, and the survival of cancer patients has increased [1]. Recent studies in Europe have estimated that more than 60% of patients survive their cancer [2]. Return to work is a significant issue, as approximately 50% of patients have jobs at the time of diagnosis [3]. In addition, return to work after treatment of cancer is an important step for patient’s social recovery. Return to work represents not only recovery of financial ability but also improvements in self-confidence.

Primary liver cancer is the fifth most common malignancy and one of the leading causes of cancer-related death worldwide [4]. To our knowledge, the most common type of primary liver cancer is hepatocellular carcinoma [5]. The incidence rate varies geographically and sexually throughout the world. Age standardized incidence rates between gender in East and South-East Asia was 21.4 ~ 35.5 per 100,000 in male and 9.0 ~ 12.7 per 100,000 in female. Whereas, in South-Central and in Northern Europe was 3.4 ~ 3.8 per 100,000 in male and 1.6 per 100,000 in female [6]. In Taiwan, the age standardized incidence rate was 54.34 per 100,000 in 2002 and 47.11 per 100,000 in 2012. Male to female ratio was 2.52 in 2002 and 2.50 in 2012 [7]. Liver cancer also results in 5000–7000 deaths per year and has become the second leading cause of cancer-related deaths in the last 20 years in Taiwan. Hepatitis B virus (HBV) or hepatitis C virus (HCV), alcohol consumption, smoking, obesity, genetic factors and aflatoxin exposure affect the incidence of liver cancer [8]. Chronic inflammation of hepatocytes causes noncirrhotic and cirrhotic changes and leads to liver cancer [9]. In recent years, due to hepatitis virus vaccine and multimodal treatment, the incidence and mortality of liver cancer has been decreasing [10]. However, there are many complications still associated with liver cancer. Impaired liver function leads to many problems, including jaundice, anemia, ascites and bleeding [11]. All of these problems limit patient quality of life and working ability. Surgical resection is the mainstay of treatment for in the early stage (carcinoma in situ) of disease. However, overall (population-based) survival is still poor for the majority of patients diagnosed in the late course and those unsuitable for curative therapy [12]. Fortunately, tremendous improvements have been made regarding drug treatment for advanced liver cancer. Oral small molecule multikinase inhibitors and monoclonal antibodies have proven efficacy as first- or second-line therapies. Furthermore, these treatments have a positive benefit on quality of life [13]. Due to improving treatment options, the number of long-term cancer survivors is increasing. Return to work has become a significant problem for the past few years.

According to literature reviews, liver cancer and return to work was only a small part of study in cancer impact and employment status. Results of these studies concluded that liver cancer was one of barriers about return to work [14, 15]. The goals of this cohort study are to discuss the relationship between return to work and various covariables, including cancer treatment, comorbidities, financial status, social characteristics and cancer stage, in workers with liver cancer. Furthermore, the effect of return to work on all-cause mortality and the survival rate of patients with liver cancer in Taiwan was also analyzed in our study.

## Methods

The data for this study were from population databases in Taiwan and included data collected from adult patients diagnosed with liver cancer in the period 2004–2010. These data were collected by the National Health Insurance Research Database (NHIRD), Labor Insurance Database (LID), and the Taiwan Cancer Registry (TCR), and the data were connected by an encrypted number. All procedures were conducted in accordance with the regulations and guidelines of the Institutional Review Board (1–107–05-129) in Tri-service General Hospital (TSGH). First, pertinent information, which included the employee industry, employment data, and working district, were extracted from the LID. Next, we connected the identification number in the LID with the TCR and NHIRD databases.

### Study sample

There were 2451 workers included in our study, and they were first diagnosed with liver cancer in the period of 2004–2010. The exclusion criteria for this analysis were being twenty years of age or younger, being unemployed at baseline, having liver cancer in combination with other cancers, and having a liver cancer diagnosis before 2004. In the 2nd year, there were 1504 workers who had gone back to work, 550 deaths, and 397 unemployed individuals; in the 5th year, there were 1123 workers who had gone back to work, 940 deaths, and 388 unemployed individuals. The demographic characteristics of workers, including age, gender, comorbidities, district of domicile, monthly income, corporation size, and the stages of liver cancer, are presented in Table 1. A total of 1123 (45.8%) patients were reemployed five years after liver cancer.

**Table 1:** Demographic data of RTW group and non-RTW group

Variables	RTW (***N*** = 1123)	Non-RTW (***N*** = 1328)	***p*** value
**Characteristic**			
**Age (years)**	50.5 ± 8.8 (23 ~ 81)	52.7 ± 9.5 (23 ~ 85)	< 0.0001
**Gender (male)**	874 (77.8%)	1068 (80.4%)	0.1146
**Comorbidities**			
**Disorders of lipoid metabolism**	100 (8.9%)	134 (10.1%)	0.3196
**Alcohol abuse**	39 (3.5%)	31 (2.3%)	0.0918
**Hypertension**	230 (20.5%)	303 (22.8%)	0.1626
**Congestive heart failure**	12 (1.1%)	21 (1.6%)	0.2724
**Peripheral vascular disease**	8 (0.7%)	15 (1.1%)	0.2859
**Cerebrovascular disease**	20 (1.8%)	39 (2.9%)	0.0629
**Chronic pulmonary disease**	53 (4.7%)	77 (5.8%)	0.2351
**Rheumatologic disease**	14 (1.2%)	10 (0.8%)	0.2162
**Peptic ulcer disease**	178 (15.9%)	241 (18.1%)	0.1323
**Mild liver disease**	704 (62.7%)	695 (52.3%)	< 0.0001
**Renal disease**	42 (3.7%)	50 (3.8%)	0.974
**Moderate or severe liver disease**	37 (3.3%)	38 (2.9%)	0.5349
**Depression**	37 (3.3%)	37 (2.8%)	0.4635
**Treatment**			
**Operation**	1100 (98.0%)	1260 (94.9%)	< 0.0001
**Radiation therapy**	6 (0.5%)	33 (2.5%)	0.0001
**Chemotherapy**	67 (6.0%)	156 (11.7%)	< 0.0001
**Living area when diagnosed of cancer**			0.5736
**Central**	227 (20.2%)	290 (21.8%)	
**North**	517 (46.0%)	626 (47.1%)	
**East**	20 (1.8%)	24 (18.2%)	
**South**	351 (31.3%)	382 (28.8%)	
**Islands**	8 (0.7%)	6 (0.5%)	
**Income range (TWD)**			
**< 28,800**	658 (58.6%)	691 (52.0%)	
**28,800–38,200**	205 (18.3%)	161 (12.1%)	
**> 38,200**	260 (23.2)	476 (35.8%)	
**Industrial classification**		0.0585	
**Agriculture**	97 (8.6%)	109 (8.2%)	
**Manufacturing**	360 (32.1%)	408 (30.7%)	
**Electricity Supply**	5 (0.4%)	18 (1.4%)	
**Water Supply**	7 (0.6%)	11 (0.8%)	
**Construction**	157 (14.0%)	188 (14.2%)	
**Wholesale**	117 (10.4%)	153 (11.5%)	
**Transportation**	111 (9.9%)	102 (7.7%)	
**Food Service**	33 (2.9%)	43 (3.2%)	
**Information**	22 (2.0%)	21 (1.6%)	
**Financial**	23 (2.0%)	28 (2.1%)	
**Real Estate**	9 (0.8%)	22 (1.7%)	
**Technical Activities**	22 (2.0%)	27 (2.0%)	
**Support Service**	21 (1.9%)	41 (3.1%)	
**Public Administration**	14 (1.2%)	25 (1.9%)	
**Education**	13 (1.2%)	17 (1.3%)	
**Health Care**	12 (1.1%)	22 (1.7%)	
**Other Service**	100 (8.9%)	93 (7.0%)	
**Company size**			0.0141
**Shut down**	89 (7.9%)	152 (11.4%)	
**Small**	80 (7.1%)	109 (8.2%)	
**Medium**	225 (20.0%)	265 (20.0%)	
**Large**	729 (64.9%)	802 (60.4%)	
**Pathological Tumor stage**			< 0.0001
**0,1**	718 (63.9%)	503 (37.9%)	
**2**	276 (24.6%)	369 (27.8%)	
**3**	105 (9.3%)	359 (27.0%)	
**4**	24 (2.1%)	97 (7.3%)	
**Pathological Node stage**		< 0.0001	
**0**	1117 (99.5%)	1266 (95.3%)	
**1**	6 (0.5%)	62 (4.7%)	
**Pathological Metastasis stage**			< 0.0001
**0**	1120 (99.7%)	1280 (96.4%)	
**1**	3 (0.3%)	48 (3.6%)	
**Pathological stage**			< 0.0001
**0,1**	715 (63.7%)	495 (37.3%)	
**2**	274 (24.4%)	347 (26.1%)	
**3**	131 (11.7%)	438 (33.0%)	
**4**	3 (0.3%)	48 (3.6%)	

### Ethical considerations

Since the study used deidentified materials from National registry database. All protocols were executed by the Institutional Review Board (1–107–05-129) of TSGH.

### Diagnosis of liver cancer

In accordance with the International Classification of Diseases for Oncology code (ICD-O-3: C22), we listed the primary site, staging, and histology of liver cancer. We used the American Joint Committee on Cancer (AJCC) 8th Edition staging system for hepatocellular carcinoma (HCC) (Table 2).

**Table 2:** AJCC 8th Edition Staging System for Hepatocellular carcinoma

Primary tumor (T)				Regional lymph nodes (N)		Distant metastases (M)	
T1a	Solitary tumor < 2 cm with/without vascular invasion			Nx	Regional lymph nodes cannot be assessed	M0	No distant metastasis
T1b	Solitary tumor > 2 cm without vascular invasion			N0	No regional lymph node metastasis	M1	Distant metastasis
T2	Solitary tumor > 2 cm with vascular invasion or multifocal tumors, none > 5 cm			N1	Regional lymph node metastasis		
T3	Multifocal tumors at least one of which is > 5 cm						
T4	Single tumor or multifocal tumors of any size involving a major branch of the portal vein or hepatic vein or tumor(s) with direct invasion of adjacent organs other than the gallbladder or with perforation of visceral peritoneum						
Stage IA	T1a	N0	M0				
Stage IB	T1b	N0	M0				
Stage II	T2	N0	M0				
Stage IIIA	T3	N0	M0				
Stage IIIB	T4	N0	M0				
Stage IVA	Any T	N1	M0				
Stage IVB	Any T	Any N	M1				

### Clinical confounder assessment

We collected age, gender, job district, monthly insured salary, and corporation scale from the LID. On the basis of the ICD-9-CM codes, comorbidities listed from the NHIRD database included obesity, lipid metabolic disorders, alcohol abuse, hypertension, congestive heart failure, rheumatologic disease, renal disorders, peptic ulcer diseases and liver diseases. All of these ICD-9-CM codes are presented in Supplement Table 1. Treatments with chemotherapy, radial therapy, and surgery and the pathological stage of liver cancer were also analyzed.

### Outcome measures

The major result in our study was the return-to-work rate 1–5 years after liver cancer diagnosis. Complete work resumption after sickness was the model of return to work in the study [16], and we used the LID database to confirm return to work. The database traced every eligible subject from the baseline assessment to the termination of follow-up or death. The associations between return to work and different cancer stage survival rates were also analyzed in our study. In addition, all-cause mortality after return to work for workers with liver cancer was the secondary endpoint.

### Statistical analysis

We used the SAS statistical software package (version 9.3, SAS Institute Inc., Cary, North Carolina) for analysis in the study. We considered two-sided 푃 values smaller than 0.05 as significant. Percentages, frequencies, means, and standard deviations were included in descriptive statistical analyses. Continuous variables were investigated with independent-sample t tests and Wilcoxon rank sum tests, and categorical variables were compared by chi-square tests. Return to work after liver cancer survival was determined to be returning to the same work or reemployment within five years after cancer diagnosis. In addition, we recorded survival time from diagnosis of liver cancer until death in the period of 2004–2010. The Cox regression model was defined as the influence of different variables on return to work and survival rate.

## Results

### Sample characteristics

Table 1 presents the clinical characteristics and demographics of participants stratified by return to work and non-return to work. The mean age of the return-to-work group was 50.5 ± 8.8 years, and the mean age of the non-return-to-work group was 52.7 ± 9.5 years. A total of 98.0% returned to work, and 94.9% of the non-return-to-work group received surgical treatment (*p* < 0.05). In addition, radiation therapy, chemotherapy, company size and pathological staging also presented statistical significance (*p* < 0.05).

### Univariate and multivariate correlations between return to work and independent variables in the 2nd year

**Fig. 1:**
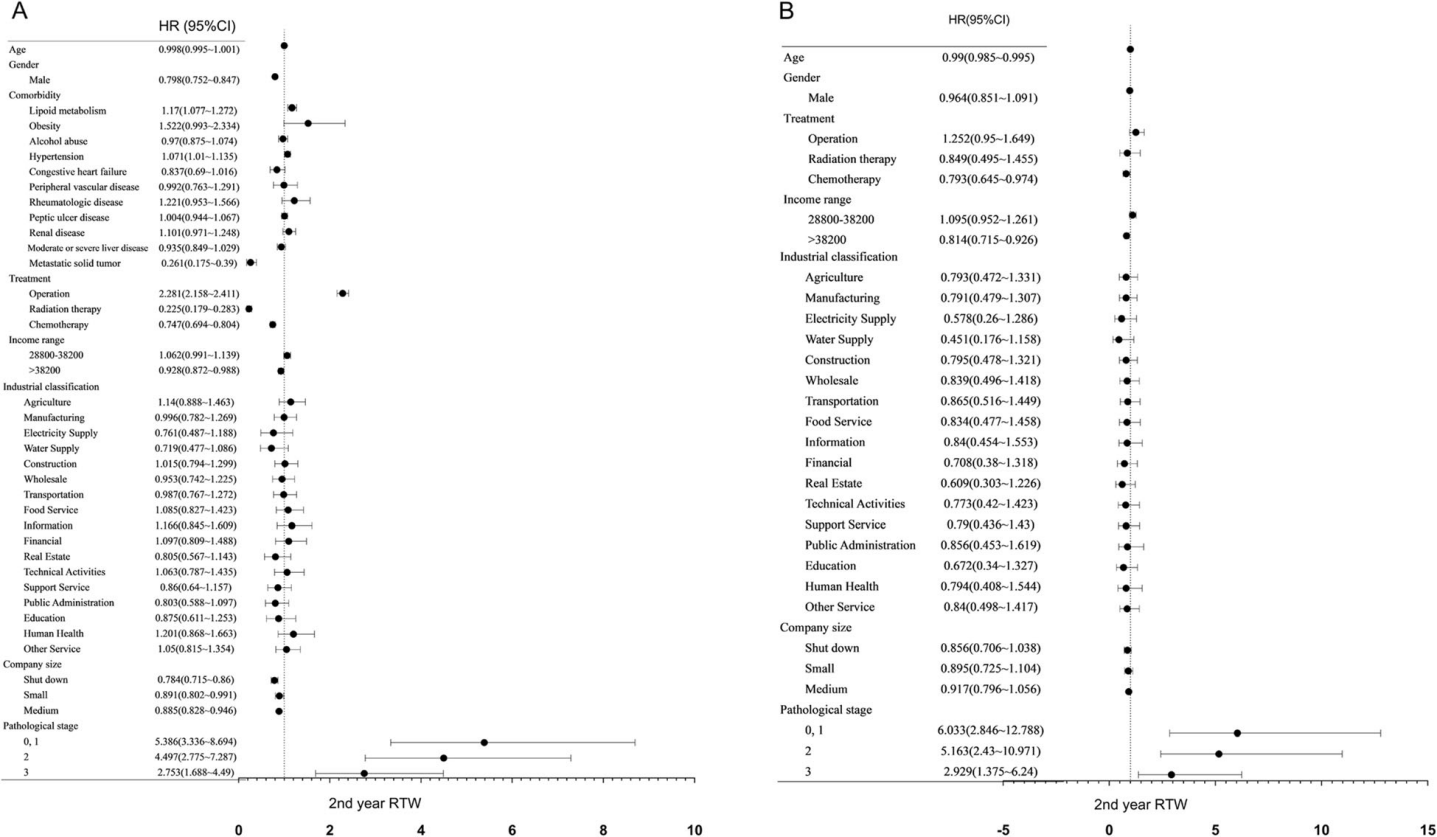
Univariate and Multivariate correlation among return to work and independent variables in the 2nd year **A** Univariate correlation **B** Multivariate correlation

In Fig. 1, we present univariate and multivariate correlations between return to work and independent variables and hazard ratios (HRs) in the 2nd year. Regarding univariate correlations, male sex, surgical treatment, lower income range and large company size were positively related to return to work (*p* < 0.05) (Fig. 1A). In the multivariate correlation, however, only young age and lower income range were positively related to return to work (*p* < 0.05) (Fig. 1B). In terms of the pathological staging, stage I to III liver cancer was positively associated with return to work in univariate and multivariate correlations.

### Univariate and multivariate correlation between return to work and independent variables in the 5th year

**Fig. 2:**
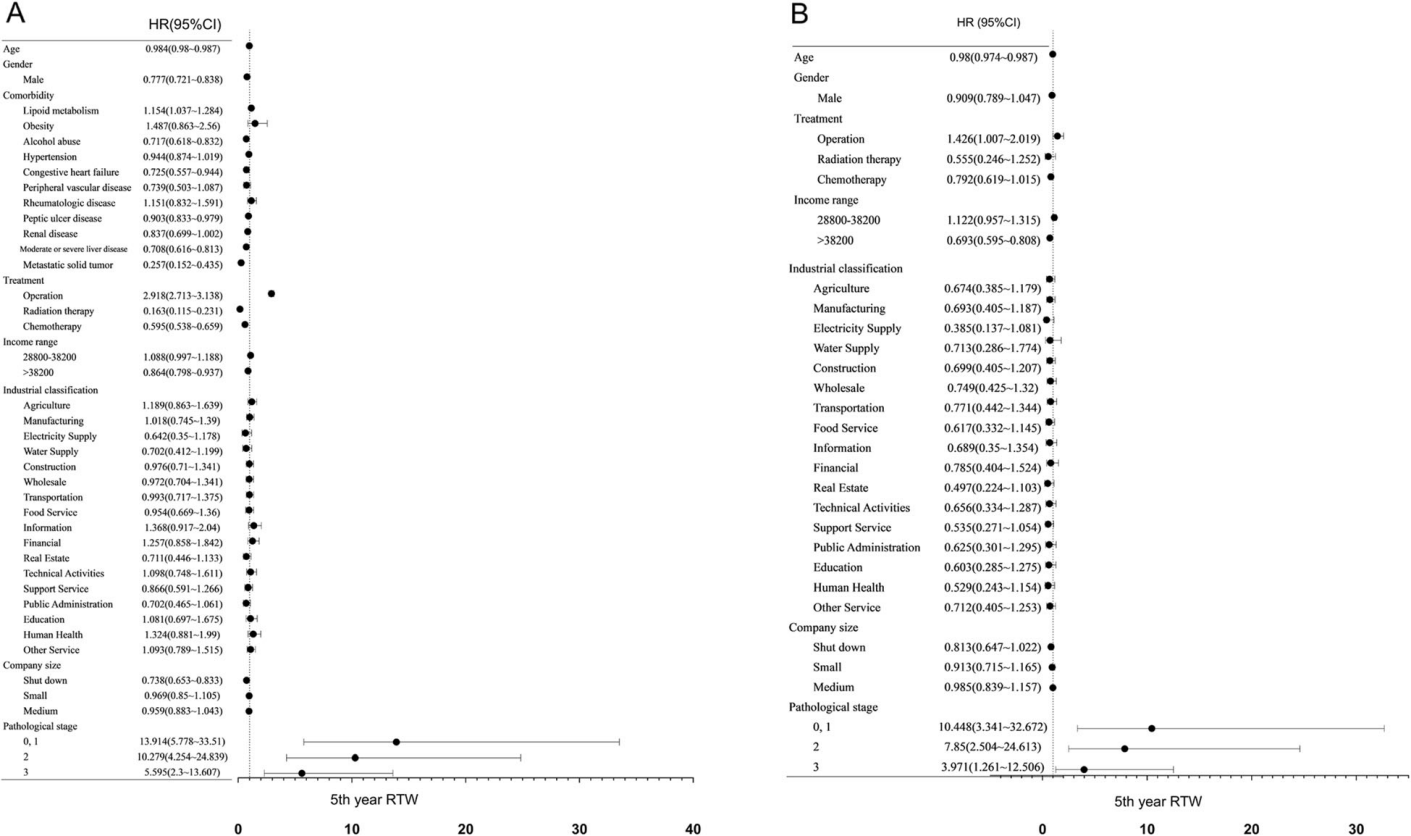
Univariate and Multivariate correlation among return to work and independent variables in the 5th year **A** Univariate correlation **B** Multivariate correlation

In Fig. 2, we present univariate and multivariate correlations between return to work and independent variables and hazard ratios (HRs) in the 5th year. Regarding univariate correlations, young age, male sex, surgical treatment, lower income range and large company size were positively related to return to work (*p* < 0.05) (Fig. 2A). In the multivariate correlation, however, only young age, surgical treatment and lower income range were positively related to return to work (*p* < 0.05) (Fig. 2B). In terms of the pathological staging, stages I to III liver cancer were positively associated with return to work in univariate and multivariate correlations.

### The association between return to work and survival outcomes in liver cancer survivors

**Fig. 3:**
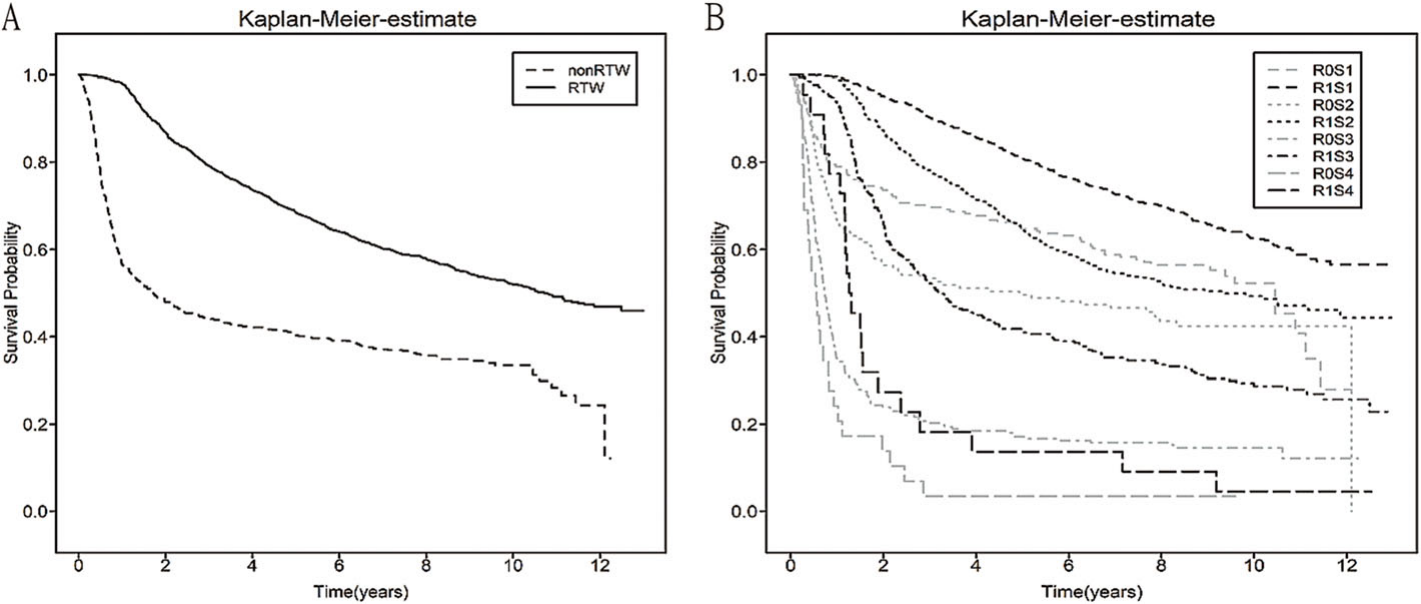
Kaplan-Meier curve for all-cause mortality categorized by different stage of liver cancer. **A** All stage of liver cancer **B** Stage 1–4 of liver cancer R0: non-RTW, R1: RTW; S0–4: stage 0–4: Pathological stage 0–4

A lower survival rate was noted in the non-return-to-work group (*p* < 0.001) in all patients with liver cancer (Fig. 3). Our analysis indicated that the non-return-to-work patients had a lower survival rate in the patients with stage I (*p* < 0.001), II (*p* = 0.0153), III (*p* < 0.001) and IV (*p* < 0.001) liver cancer (Fig. 3B). Moreover, regarding all-cause mortality, the return-to-work group had a higher survival rate (*p* < 0.001) (Fig. 3A). The unadjusted and fully adjusted model identified that the rate of return to work was related to all-cause mortality with an HR of 0.244 (95% CI: 0.235–0.253) and 0.434(95% CI: 0.383–0.492) (Supplement Table 2). All of these data illustrated that return to work was associated with a higher survival rate and decreased all-cause mortality in patients with liver cancer.

## Discussion

In our study, we analyzed the independent effects of cancer treatment, comorbidities, financial status, social characteristics and cancer stage on return to work from the 1st to 5th year after liver cancer diagnosis in survivors. We demonstrated that surgical treatment had positive effect for patients with liver cancer and increased the rate of 5th-year return to work. In contrast, chemotherapy was associated with a lower rate of 2nd-year return to work. In social characteristics, the rate of return to work had no differences between industries but higher in large companies. Interestingly, our study noted that lower income group (< 28800NTD) had higher rate of return to work. In addition, we also found that the lower survival rate was noted in liver cancer patients who did not return to work.

Based on the Barcelona Clinic Liver Cancer (BCLC) staging system, surgical treatment usually performed for patients with early stage (such as carcinoma in situ and Child-Pugh A) [17]. In contrast, chemotherapy is usually used in advanced hepatocellular carcinoma, which is not an applicable curative treatment. Compared with the rate of return to work in pathological stage, we can reasonably understand that surgical treatment and in the early stages of cancer are related to higher rate of return to work. The side effects of chemotherapy, such as neutropenia, neuropathy, edema, nausea, vomiting, and fatigue would decrease not only tolerability but also working capacity [18]. It was not surprisingly that the chemotherapy and late-stage liver cancer survivors showed a lower rate of return to work in the 2nd and 5th years in our study.

Comorbidities also play an important role in our study. The lower rate of 5th year return to work was associated with metastatic solid tumor, alcohol consumption and heart failure. The patients with metastatic solid tumor mean the patients at the end-stage of cancer. Alcohol consumption connected with liver cirrhosis increasing the severity of comorbidities such as portal hypertension, ascites, varices, infection and encephalopathy [19]. Heart failure is also related to the cardiotoxicity of chemotherapy, which increases mortality in liver cancer survivors [20]. All of these comorbidities have been shown to increase the rate of mortality in liver cancer survivors.

The work environment, culture, and resources are the main reasons for different work performances. A previous study demonstrated that lower educational level and poorer perceived financial status were related to delayed return to work in colorectal cancer survivors [21]. In our study, we used industrial classification to represent the difference in work environment, but no associations were noted between different industries and the rate of return to work in liver cancer survivors. We used company size to indicate the work environment and resources in workplace. The rate of return to work was higher in large companies than small ones in the 2nd year. Generally, a large company provides a better work environment and health care for its employees than a small enterprise [22]. These complete institutions provided not only financial support but also occupational counseling and a friendly environment for cancer survivors.

Different from other research, in our study, the rate of return to work was higher in lower income range group. For these conflicting results, we established two hypotheses. First, lower income range group had more intent to return to work due to economic pressure. Second, compared the national health insurance system with other countries, Taiwan’s health insurance system covers nearly 100% of the population [23] and provides comprehensive medical services. Annually, gross domestic product (GDP) proportion spent on health was about 6.4% in Taiwan [24]. It means that every citizen only needs to pay a small fee to maintain their medical security. Due to these features of Taiwan’s national health insurance system, we supposed that lower income group had strong incentive to return to work.

The survivors return to work has very significant meaning for patients and society. It is related to having a purpose of life, a stable income, and a sense of contributing [25]. In other words, they had functional recovery. The degrees of functional recovery were widely used to estimate the quality of life in different diseases. Cancer survivors who return to work had engaged in more physical activities because they found increasing value in their work and less lassitude, and all of these factors might contribute to an increase in their survival rate [26]. A study of hepatocellular carcinoma in Sweden showed that patient-reported quality of life was prognostic for overall survival [27]. These findings were identical with our study and further support that return to work had a higher survival rate than those who remained unemployed or on sick leave in liver cancer survivors. Although return to work has increased the survival rate of liver cancer survivors, reducing work engagement and work abilities among cancer survivors was another nonnegligible issue.

This study had both strengths and limitations. The strength of the current analysis was that a large population was included in our study. Moreover, we examined many confounders and discussed their connection with exposure and outcome. Unfortunately, there were still some limitations in our study. First, we did not include HBV and HCV infection data in our analysis. The impact of HBV and HCV infection on liver cancer has been examined in many studies. Second, educational level was not presented in this study, but educational level has been shown to strongly impact return to work [28]. Finally, our current study used the NHIRD and LID databases, which came from the national cancer screening project. Thus, causal inferences are complicated due to environmental and occupational effects.

## Conclusions

Our study identified the impacts of medical and sociodemographic factors on the return to work of liver cancer survivors. In addition, in patients with liver cancer, the return to work had a positive effect on the survival rate. Side effects of disease or treatment, including physical and psychological problems, also affected patients’ working abilities and the rate of return to work. In summary, disease, reemployment, treatment and survival rate were inseparable and interfered with each other for patients with liver cancer.

## Supplementary Information

**Additional file 1: Supplement Table 1.** ICD-9-CM codes of comorbidities listed from the NHIRD database.

**Additional file 2: Supplement Table 2.** Associations between the return to work and all caused mortality.

## Data Availability

Raw data were generated at National Health Insurance Research Database and Labor Insurance Database in Taiwan. Derived data supporting the findings of this study are available from the National Health Insurance Administration, Ministry of Health and Welfare in Taiwan, Republic of China (R. O. C.). (URL: https://dep.mohw.gov.tw/dos/lp-5146-113.html).

## Abbreviations

HBV: Hepatitis B virus
HCV: Hepatitis C virus
NHIRD: National Health Insurance Research Database
LID: Labor Insurance Database
TCR: Taiwan Cancer Registry
TSGH: Tri-service general hospital
ICD: International Classification of Diseases
AJCC: American Joint Committee on Cancer
HCC: Hepatocellular carcinoma
BCLC: Barcelona Clinic Liver Cancer
