# Supplementary material for: Investigation on returning to work in liver cancer survivors in Taiwan: a 5-year follow-up study
Source: BMC Public Health. 2021 Oct 12;21:1846. doi: 10.1186/s12889-021-11872-9 (PMC8507177; doi:10.1186/s12889-021-11872-9)
Supplement: Supplementary file 1 — Additional file 1: Supplement Table 1. ICD-9-CM codes of comorbidities listed from the NHIRD database. [file 12889_2021_11872_MOESM1_ESM.docx]

| Comorbidities | ICD-9-CM codes |
| --- | --- |
| Obesity | 278.00, 278.01, 278.02 |
| Lipid metabolic disorders | 272.0-272.9 |
| Alcohol abuse | 265.2, 291.1-291.3, 291.5-291.9, 303.0, 303.9, 305.0, 357.5, 425.5, 535.3, 571.0-571.3, 980.x, V11.3 |
| Hypertension | 401.0, 401.1, 401.9 |
| Congestive heart failure | 428.0 |
| Peripheral vascular disease | 443.0, 443.1, 443.21-443.24, 443.29, 443.81, 443.82, 443.89, 443.9 |
| Rheumatologic disease | 719.3x, 725.x-728.x, 729.0 |
| Renal disorders | 403.01, 403.11, 403.91, 404.02, 404.03, 404.12, 404.13, 404.92, 404.93, 582.x, 583.0-583.7, 585.x, 586.x, V42.0, V45.1, V56.x |
| Peptic ulcer diseases | 531.x-534.x |
| Liver diseases | 070.22, 070.23, 070.32, 070.33, 070.44, 070.54, 070.6, 070.9, 570.x, 571.x, 573.3, 573.4, 573.8, 573.9, V42.7 |

Supplement table 1: ICD-9-CM codes of comorbidities listed from the NHIRD database
