# Supplementary material for: Investigation on returning to work in liver cancer survivors in Taiwan: a 5-year follow-up study
Source: BMC Public Health. 2021 Oct 12;21:1846. doi: 10.1186/s12889-021-11872-9 (PMC8507177; doi:10.1186/s12889-021-11872-9)
Supplement: Supplementary file 2 — Additional file 2: Supplement Table 2. Associations between the return to work and all caused mortality. [file 12889_2021_11872_MOESM2_ESM.docx]

Supplement table 2. Associations between the return to work and all caused mortality

|  | Unadjusted  HR (95% CI) | P Value | Fully adjusted  HR (95% CI) | P Value |
| --- | --- | --- | --- | --- |
| Return to work | 0.244(0.235-0.253) | <0.0001 | 0.434(0.383-0.492) | <0.0001 |

Adjusted covariates: age, treatment, income range, industrial classification, company size, cancer stage
